# Effect on Insulin-Stimulated Release of D-Chiro-Inositol-Containing Inositolphosphoglycan Mediator during Weight Loss in Obese Women with and without Polycystic Ovary Syndrome

**DOI:** 10.1155/2016/7631804

**Published:** 2016-09-18

**Authors:** Kai I. Cheang, Sakita N. Sistrun, Kelley S. Morel, John E. Nestler

**Affiliations:** ^1^Department of Pharmacotherapy & Outcomes Science, School of Pharmacy, Virginia Commonwealth University, P.O. Box 980533, Richmond, VA 23298-0533, USA; ^2^Virginia Commonwealth University Institute for Women's Health, P.O. Box 980319, Richmond, VA 23298-0319, USA; ^3^Bionutrition Service, Center for Clinical and Translational Research, Virginia Commonwealth University, P.O. Box 980155, Richmond, VA 23298-0155, USA; ^4^Carilion Clinic Obstetrics & Gynecology, 102 Highland Ave, Suite 455, Roanoke, VA 24013, USA; ^5^Division of Endocrinology and Metabolism, Department of Internal Medicine, School of Medicine, Virginia Commonwealth University, P.O. Box 980111, Richmond, VA 23298-0111, USA

## Abstract

*Background.* A deficiency of D-chiro-inositol-inositolphosphoglycan mediator (DCI-IPG) may contribute to insulin resistance in polycystic ovary syndrome (PCOS). Whether the relationship between impaired DCI-IPG release and insulin resistance is specific to PCOS rather than obesity is unknown. We assessed insulin-released DCI-IPG and its relationship to insulin sensitivity at baseline and after weight loss in obese women with and without PCOS.* Methods.* Obese PCOS (*n* = 16) and normal (*n* = 15) women underwent 8 weeks of a hypocaloric diet. The Matsuda index, area under the curve DCI-IPG (AUC_DCI-IPG_), AUC_insulin_, and AUC_DCI-IPG_/AUC_insulin_ were measured during a 2 hr OGTT at baseline and 8 weeks.* Results.* PCOS women had lower AUC_DCI-IPG_/AUC_insulin_ at baseline and a significant relationship between AUC_DCI-IPG_/AUC_insulin_ and Matsuda index (*p* = 0.0003), which was not present in controls. Weight loss was similar between PCOS (−4.08 kg) and normal women (−4.29 kg, *p* = 0.6281). Weight loss in PCOS women did not change the relationship between AUC_DCI-IPG_/AUC_insulin_ and Matsuda index (*p* = 0.0100), and this relationship remained absent in control women.* Conclusion.* The association between AUC_DCI-IPG_/AUC_insulin_ and insulin sensitivity was only found in PCOS but not in normal women, and this relationship was unaffected by weight loss. DCI and its messenger may contribute to insulin resistance in PCOS independent of obesity.

## 1. Introduction

Polycystic ovary syndrome (PCOS) affects approximately 6–10% of women of reproductive age [1]. The disorder is characterized by chronic oligo- or anovulation and biochemical or clinical androgen excess. PCOS is also associated with increased risk for diabetes, metabolic syndrome, and early cardiovascular disease. Insulin resistance and its compensatory hyperinsulinemia play a central role in the pathogenesis of PCOS [2, 3]. Multiple lines of evidence indicate that a putative inositolphosphoglycan (IPG) second messenger, D-chiro-inositol-inositolphosphoglycan mediator (DCI-IPG), may mediate insulin action [4]. A deficiency of DCI-IPG may contribute to insulin resistance in individuals with Type 2 diabetes [5] as well as women with PCOS [6, 7]. Interventional studies with oral administration of DCI reported decreases in serum insulin and androgen levels, as well as improved ovulatory function in obese women with PCOS [8–10]. Conversely, administration of insulin sensitizers such as metformin [7] and pioglitazone [11] also increases insulin-stimulated release of DCI-IPG in women with PCOS.

Our group has previously demonstrated that the coupling between insulin action and release of DCI-IPG mediator is impaired in* obese* women with PCOS, as compared to* nonobese* normal women, suggesting that the insulin-stimulated release of bioactive DCI-IPG mediator is defective in PCOS women with obesity [12]. However, whether the relationship between impaired DCI-IPG mediator release and insulin resistance is specific to PCOS status or due to obesity per se is unknown. We hypothesize that the insulin-stimulated DCI-IPG mediator modulates insulin sensitivity in women with PCOS but not in normal women, and a reduction in obesity does not affect this relationship. To test this hypothesis, we conducted a pilot weight loss intervention study in obese women with PCOS and obese normal women. We assessed the release of both insulin and DCI-IPG mediator during an oral glucose tolerance test (OGTT), as well as insulin sensitivity as measured by the Matsuda index, at baseline and after 8 weeks of a hypocaloric diet in obese women with and without PCOS.

## 2. Materials and Methods

### 2.1. Participants

This study was performed at the Clinical Research Service Unit of Virginia Commonwealth University's Center for Clinical and Translational Research. The study was approved by the Virginia Commonwealth University Institutional Review Board. All study participants provided informed consent.

Women in this study were obese (≥30 kg/m^2^) and between the ages of 18 and 40 years. PCOS was defined by the modified Rotterdam criteria, after excluding other endocrine disorders [13]. In this study, all PCOS women had biochemical hyperandrogenemia and oligo- or amenorrhea (eight or few menstrual periods annually). Secondary causes of hyperandrogenemia or ovulatory dysfunction were excluded by normal thyroid function tests and serum prolactin and a fasting 17*α*-hydroxyprogesterone <200 ng/dL. The control group consisted of regular cycling women with normal serum testosterone. The exclusion criteria for all women included weight loss attempts by either diet or exercise within 3 months of study participation, diabetes mellitus by fasting glucose or oral glucose tolerance test (OGTT), clinically significant pulmonary, cardiac, renal, hepatic, neurologic, psychiatric, infectious, neoplastic, and malignant disease, or pregnancy as documented by urine hCG. PCOS women with disorders associated with insulin resistance, for example, hypertension or dyslipidemia, were not excluded as long as they had been on a stable dose of medication for 6 months. Normal women were excluded if they had a history of gestational diabetes or had a first-degree relative with diabetes or if they demonstrated abnormal glucose tolerance at baseline or if they had hypertension or dyslipidemia.

### 2.2. Study Procedures

PCOS women were studied during the equivalent of the follicular phase of the cycle, and normal women were studied during the mid-follicular phase of the menstrual cycle (days 5–9), as documented by a serum progesterone ≤2 ng/mL.

Because DCI may be ingested in a diet high in legumes or fruits, all subjects were interviewed by a dietician to identify those who may be consuming diets containing unusually high amounts of inositols. All participants were given instructions for a balanced mixed diet to be followed for at least three days prior to each study visit.

On the study day, the participants arrived at the Clinical Research Service Unit at Virginia Commonwealth University at 08:00 h after a 12-hour fast. Height and weight were measured to the nearest 0.1 cm and 0.1 kg using a precision stadiometer and digital scale. Waist was measured at the level of the umbilicus, and hip circumference was measured at the widest diameter of the buttocks to the nearest 0.1 cm. Fasting blood samples were drawn at 08:15, 08:30, and 08:45 h and pooled for determination of fasting insulin, glucose, and sex steroids (testosterone). At 09:00 h, an OGTT was performed by administering 75 g oral glucose. Blood samples for plasma glucose, insulin, and DCI-IPG were collected every 15 minutes for 2 hours.

After glucose and DCI assessments, the participants met with a study dietician for instruction on a hypocaloric diet. A diet-overview handout, instructional nutrition labels, sample menus and recipes, and a book on counting calories were provided. The women were instructed to follow an 8-week course of standardized hypocaloric diet containing 50% carbohydrates, 30% total lipids, and 20% proteins. They were instructed to maintain these hypocaloric diets by caloric restriction to create a deficit of 500–1000 kcal/day, as per obesity management guidelines of the National Heart, Lung, and Blood Institute [14]. This hypocaloric diet has been shown to yield weight loss of about 1 to 2 lbs/week [14]. The women were instructed specifically to avoid making any conscious effort to modify physical activity or attempt other weight loss methods in addition to the hypocaloric diets per this protocol. This is because physical activity improves insulin sensitivity even in the absence of substantial weight loss [15] and will confound our investigation of the effect of weight reduction in DCI handling and insulin sensitivity in these women. During this 8-week period, the participants purchased and prepared their own meals and maintained daily food logs. They attended follow-up visits once weekly for weight measurements. During these weekly visits, they submitted their food logs and received follow-up consultations with the study dietician.

The women returned for DCI and insulin sensitivity measurements after 8 weeks of dietary intervention. After confirmation that they were in the equivalent of the follicular phase of the menstrual cycle by serum progesterone, all measurements and testing performed at baseline (anthropometric measurements, OGTT, and blood sampling) were repeated.

### 2.3. Laboratory Analyses

Serum and plasma were stored at −80°C until being assayed. Serum glucose was measured by glucose oxidative method (YSI 2300 Stat Plus Glucose Analyzer; Yellow Springs Instruments). Serum insulin levels were measured by enzyme-linked immunosorbent assay (ELISA) (Alpco Diagnostics, Salem NH). Serum testosterone and sex hormone binding globulin (SHBG) were measured via ELISA (Alpco Diagnostics). Free testosterone was calculated using the method of Södergard et al. [16]. DCI-IPG bioactivity was measured using an in-house bioactivity assay developed by the laboratory of JEN, as previously described [7].

### 2.4. Statistical Analysis

We examined the response of serum insulin concentrations and the relative bioactivity of DCI-IPG to the oral administration of glucose by calculating the areas under the respective response curves (AUC) by the trapezoidal rule. Since insulin is thought to mediate the release of DCI-IPG after a glucose load [17] and there are interparticipant variations in AUC_insulin_, the ratio of AUC_DCI-IPG_/AUC_insulin_ more accurately reflects insulin-mediated release of DCI-IPG than AUC_DCI-IPG_ alone. Hence, we used this ratio in our analyses. Whole body insulin sensitivity as described by Matsuda and DeFronzo [18] was used to assess insulin sensitivity.

Comparisons between groups at baseline were made with Student's two-tailed *t*-test. To assess within group effects from baseline to after treatment, a matched pairs two-tailed *t*-test was performed. To assess the treatment effects between groups, the changes in each variable (after weight loss minus baseline) were compared using a two-tailed *t*-test. Pearson's correlation was used to assess the association between change in Matsuda index and change in bioactive DCI-IPG released per unit of insulin during OGTT, after linearity and normality of residuals were assessed.

Distribution of the data was assessed by normal quantile plots. Variables not in normal distribution were log-transformed for analyses and then backtransformed into their original units for reporting. Data were presented as mean ± standard deviation or geometric mean (95% confidence interval [CI]) for parameters that were transformed for analyses. *p* < 0.05 was considered statistically significant. Analyses were performed by JMP 12.0 (SAS Institute, NC).

## 3. Results

A total of 80 women provided consent to participate. Of these, 19 met exclusion criteria before study entry. Of the remaining 34 PCOS and 27 normal women, 18 PCOS and 12 normal women dropped out prior to the follow-up visit. Hence, 16 PCOS and 15 normal women completed the study. The attrition rate in this study was similar to that of other dietary-based weight loss studies [19]. Because the purpose of this study is to evaluate the relationship between changes in DCI-IPG mediator release and changes in insulin sensitivity during weight loss in PCOS as compared to normal women, we only included women who completed the study.

### 3.1. Baseline Characteristics

At baseline, control women and women with PCOS did not differ in terms of age, racial mix, BMI, or waist-to-hip ratio (Table 1). As expected, PCOS women tended to have significantly higher serum total testosterone. Although women with PCOS had higher AUC_glucose_ and AUC_insulin_ and lower whole body insulin sensitivity as determined by Matsuda index, these differences did not attain statistical significance.

**Figure 1:**
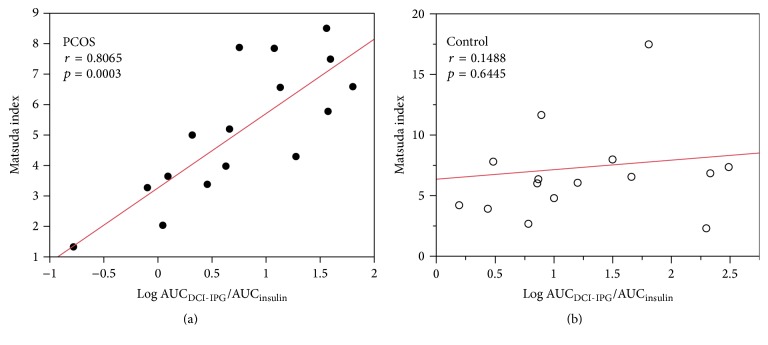
Relationship between baseline Matsuda index and release of the bioactive DCI-IPG messenger per unit of insulin released during OGTT in PCOS (●, a) and normal (⚪, b) women. DCI-IPG, D-chiro-inositol-inositolphosphoglycan mediator.

At baseline, women with PCOS had significantly lower AUC_DCI-IPG_/AUC_insulin_ ratios. In PCOS women, there was a significant relationship between AUC_DCI-IPG_/AUC_insulin_ and Matsuda index (*r* = 0.8065, *p* = 0.0003, Figure 1(a)). This relationship was not found in control women (*r* = 0.1488, *p* = 0.6445, Figure 1(b)).

### 3.2. Changes in Insulin, Glucose, and the Bioactivity Profiles of DCI-IPG after Weight Loss

After the weight loss intervention, both PCOS (−4.08 ± 3.65 kg, *p* = 0.0013) and control women (−4.69 ± 2.98 kg, *p* = 0.0005) lost weight compared to baseline. The amount of weight loss did not differ between the groups (*p* = 0.6281) (Table 2). However, the Matsuda index improved significantly only in normal women (from 6.94 ± 4.06 to 9.53 ± 4.79, *p* = 0.0479) but not in PCOS women (from 5.22 ± 2.18 to 5.39 ± 2.52, *p* = 0.8209). Weight loss did not significantly increase AUC_DCI-IPG_/AUC_insulin_ from baseline in either group (*p* = 0.6387 in PCOS and *p* = 0.9697 in normal women).

### 3.3. Relationship between DCI-IPG Mediator Bioactivity and Insulin Sensitivity after Weight Loss

**Figure 2:**
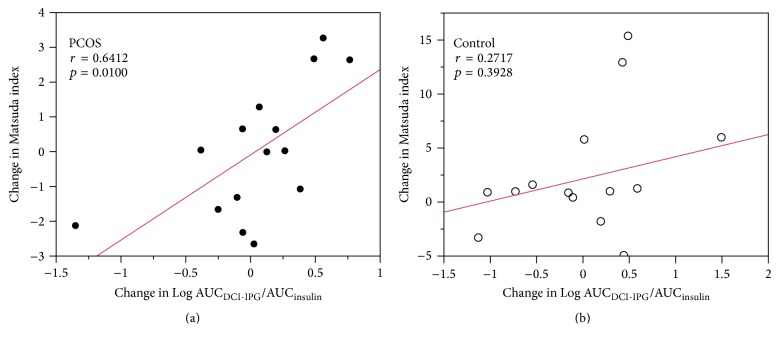
Relationship between change in Matsuda index and change in release of the bioactive DCI-IPG messenger per unit of insulin released during OGTT in PCOS (●, a) and normal (⚪, b) women after 8 weeks of weight loss intervention. DCI-IPG, D-chiro-inositol-inositolphosphoglycan mediator.

Weight loss did not change the relationship between AUC_DCI-IPG_/AUC_insulin_ and Matsuda index in PCOS women. Among women with PCOS, after weight loss there remained a significant relationship between change in AUC_DCI-IPG_/AUC_insulin_ and change in Matsuda index (*r* = 0.6412, *p* = 0.0100, Figure 2(a)). This relationship was not found in control women (*r* = 0.2717, *p* = 0.3928, Figure 2(b)).

## 4. Discussion

In this study, we observed that obese women with PCOS, as compared to normal women with similar BMI, have decreased insulin-released DCI-IPG mediator during an OGTT. We observed that the relationship between insulin sensitivity as measured by the Matsuda index and AUC_DCI-IPG_/AUC_insulin_ was found only in obese women with PCOS and not in obese normal women. Furthermore, this relationship was unaffected by weight loss. After a similar amount of weight loss, a significant relationship between AUC_DCI-IPG_/AUC_insulin_ and Matsuda index remained only in women with PCOS but was not present in normal women.

The findings of our study are in concordance with our previous report of significantly lower AUC_DCI-IPG_/AUC_insulin_ ratios in PCOS women as compared to normal women [6]. However, in the previous study, PCOS participants had a significantly higher BMI (33.9 kg/m^2^) than normal women (25.6 kg/m^2^, *p* = 0.002). Our current study demonstrates that, even with similar obesity, AUC_DCI-IPG_/AUC_insulin_ remained lower in PCOS women compared to normal women (*p* = 0.0377, Table 1). Our findings suggest that bioactivity of the DCI-IPG mediator is decreased in PCOS independent of obesity.

We also observed that the relationship between insulin sensitivity and AUC_DCI-IPG_/AUC_insulin_ was present only in women with PCOS (Figure 1(a)) and not in normal women (Figure 1(b)) and that this finding remained evident after weight loss (Figures 2(a) and 2(b)). These results are supported by our previous findings of a significant association between change in insulin sensitivity and change in DCI-IPG released per unit of insulin with oral DCI administration in PCOS women [20]. However, previous studies only examined this association in women with PCOS, and whether AUC_DCI-IPG_/AUC_insulin_ is correlated with insulin sensitivity in normal women with similar BMI has been unknown. To the authors' knowledge, this is the first report suggesting that DCI-IPG mediator release may not play a major role in insulin sensitivity in normal obese women.

Weight loss did not affect the relationship between insulin sensitivity and AUC_DCI-IPG_/AUC_insulin_ in obese women with PCOS in this study. This finding is in line with our previous reports supporting that DCI deficiency in PCOS may be unrelated to adiposity. To wit, administration of oral DCI improved AUC_insulin_, serum androgens, and ovulation to both obese [8] and lean [9] women with PCOS.

In this current study, weight loss did not significantly improve AUC_DCI-IPG_/AUC_insulin_ in either PCOS or normal women. In contrast, previous studies demonstrated that insulin sensitizers such as metformin [7] and rosiglitazone [11] improved the availability of DCI-IPG mediator release in women with PCOS.

Why do insulin sensitizers, but not weight loss as described in this study, improve the AUC_DCI-IPG_/AUC_insulin_ bioactivity profile in women with PCOS? One reason could be that weight loss of more than 4 kg in this study did not improve insulin sensitivity in obese women with PCOS. At first glance, our results seem to contradict previous research supporting the role of weight loss in improving insulin sensitivity in PCOS [21]. However, there is tremendous heterogeneity in the effect of weight loss on improving insulin sensitivity and other features of PCOS [22, 23]. In one weight loss study, as many as 50% of PCOS women did not have improved insulin sensitivity as measured by HOMA and, commensurably, no improvement in menstrual cyclicity, despite similar fat losses in both responders and nonresponders [23].

We did not observe a difference in the amount of weight loss between women with and without PCOS in this study. There have been conflicting reports about the role of insulin resistance in the regulation of obesity. Some studies suggested insulin resistance predicted weight gain [24], more weight loss [25], or no effect on weight loss [26] in obese individuals. Hence, the knowledge that weight loss is not different between PCOS and normal women when given the same hypocaloric diet can be reassuring to women with PCOS who are attempting weight loss.

A strength of this study includes similar BMI between PCOS and normal women, which helped elucidate that the relationship between AUC_DCI-IPG_/AUC_insulin_ bioactivity and insulin sensitivity is specific to PCOS and not obesity. These results are novel since the roles of DCI-IPG mediator in normal obese women have not been previously explored.

A weakness of the study is that the amount of weight loss achieved in both groups of women may have been inadequate to illicit changes in DCI-IPG/insulin ratio. Although the amount of weight loss (0.5 kg or approximately 1 lb per week) was in accordance with current weight management guidelines [14], over the course of the 8-week study period, it resulted in a reduction in weight by about 4 kg in both groups, which was less than that achieved in other weight loss studies in PCOS [23, 27]. A study with a longer duration would have resulted in a bigger magnitude in weight reduction.

In conclusion, this study demonstrated that obese women with PCOS, as compared to normal women with similar BMI, have decreased insulin-released DCI-IPG mediator during OGTT. The relationship between insulin sensitivity and AUC_DCI-IPG_/AUC_insulin_ is only found in women with PCOS but not in normal women. Furthermore, this relationship is unaffected by weight loss. After a similar amount of weight loss, a significant relationship between AUC_DCI-IPG_/AUC_insulin_ and Matsuda index is only found in women with PCOS but not in normal women. Combined with previous studies of oral DCI administration in PCOS women by our group and others, this study reinforces the contribution of DCI and its messenger in its role in insulin resistance in women with PCOS independent of obesity.

## Figures and Tables

**Table 1:** Baseline characteristics and serum hormone concentrations.

Parameter	PCOS (*n* = 16)	Control (*n* = 15)	*p* value
Age (years)	26.9 (4.6)	27.5 (5.7)	0.7267
Race			0.5649
African-American	7	7	
Caucasian	8	7	
Other	1	1	
Weight (kg)	99.2 (13.3)	97.6 (15.4)	0.7508
BMI (kg/m^2^)	36.6 (5.1)	35.8 (4.8)	0.6507
Waist circumference (cm)	101.6 (12.7)	99.9 (9.9)	0.6860
Waist/hip ratio	0.82 (0.05)	0.80 (0.08)	0.4102
Total testosterone (ng/dL)	63.1 (34.4)	27.7 (8.0)	0.0006
Free testosterone (ng/dL)	0.90 (0.87)	0.44 (0.25)	0.0786
Fasting insulin (mg/dL)^*∗*^	7.58 (5.65–10.15)	8.12 (5.06–13.03)	0.7897
Fasting glucose (mg/dL)	85.5 (8.1)	84.2 (3.8)	0.6000
AUC insulin (min·mg/dL)	7999 (5058)	5667 (2890)	0.1323
AUC glucose (min·mg/dL)	14781 (3016)	12963 (2245)	0.0715
Matsuda index	5.22 (2.18)	6.94 (4.06)	0.1577
AUC DCI-IPG (% min)	15279 (6030)	21441 (19376)	0.2539
Ratio of AUC DCI-IPG/AUC insulin (%/*µ*IU/mL)^*∗*^	2.76 (1.79–3.73)	4.83 (2.37–7.29)	0.0377

Values are mean (SD) or geometric mean (95% confidence interval) when indicated by *∗*.

**Table 2:** Changes in metabolic parameters after 8 weeks of weight loss intervention in women with and without PCOS.

Parameter	PCOS (*n* = 16)	Control (*n* = 15)	*p* value
Weight (kg)	−4.08 (−2.13, −6.02)^a^	−4.69 (−2.89, −6.49)^a^	0.6281
BMI (kg/m^2^)	−1.46 (−0.72, −2.20)^a^	−1.80 (−1.14, −2.45)^a^	0.4829
Waist/hip ratio	−0.014 (−0.034, 0.006)	−0.040 (−0.107, 0.027)	0.3878
Fasting insulin (mg/dL)	+1.8 (−2.2, +5.7)	−5.0 (−10.6, +0.6)	0.0625
Fasting glucose (mg/dL)	−1.15 (−4.43, +2.13)	+0.21 (−2.35, +2.77)	0.5041
AUC insulin (min·mg/dL)	−1009 (−2601, +642)	−1403 (−2886, −80)^b^	0.1961
AUC glucose (min·mg/dL)	−961 (−1944, +21)	+152.8 (−1245, +1550)	0.1600
Matsuda index	+0.17 (−0.87, +1.21)	+2.60 (+0.38, +5.58)^c^	0.1168
AUC DCI-IPG (% min)	−446 (−4078, +3185)	−3754 (−11496, +3989)	0.6765
Ratio of AUC DCI-IPG/AUC insulin (%/*µ*IU/mL)	+1.085 (−0.709, 1.663)	+1.049 (−0.798, 1.380)	0.8805

Data are expressed as mean (95% confidence interval).

^a^ *p* < 0.002 for within group difference between baseline and after weight loss.

^b^ *p* = 0.0134 for within group difference between baseline and after weight loss.

^c^ *p* = 0.0479 for within group difference between baseline and after weight loss.
